# Exploring AI-personalized visualization of the safe place in virtual reality versus imagination of the safe place for stress, burnout, and relaxation in psychotherapists: a case series

**DOI:** 10.3389/fdgth.2025.1665457

**Published:** 2025-11-20

**Authors:** Viktoria Wollweber, Franziska Pfannerstill, Thomas Probst

**Affiliations:** Division of Psychotherapy, Department of Psychology, University of Salzburg, Salzburg, Austria

**Keywords:** virtual reality, imagination, relaxation, burnout, psychotherapists, workplace interventions

## Abstract

**Objectives:**

Psychotherapists face substantial occupational stress and emotional demands placing them at risk of burnout, yet interventions aimed at enhancing their well-being remain understudied. This case series explored the effectiveness of a brief relaxation intervention comparing the traditional safe place imagination exercise with a virtual reality (VR) adaptation. The VR adaptation comprised a personalized visualization of the safe place by artificial intelligence (AI). The exploratory research questions investigated how both interventions would influence relaxation, burnout and stress over the course of the study and whether VR would yield different effects than imagination. Further, the roles of imagery ability and presence were explored.

**Methods:**

Five psychotherapists from an outpatient setting participated in a six-week, within-subject study that used an alternating treatments design (ABCABC) which was created via a participatory approach where participants actively engaged in the planning of the study. Imagination- (B) and VR-based (C) relaxation interventions alternated weekly with baseline weeks (A) in between. The VR environments were tailored to participants' preferences through generative AI.

**Results:**

Relaxation consistently increased in both conditions based on self-report and physiological (skin conductance levels) measures. Burnout and stress did not significantly decrease over time and VR did not yield significantly greater effects on all outcomes. Imagery ability was not associated with greater relaxation, whereas presence showed a positive correlation in one VR session.

**Conclusion:**

These findings suggest that brief imagination and VR interventions can enhance relaxation in psychotherapists. The lack of significant differences between conditions suggests that VR does not inherently outperform traditional methods, especially with participants already familiar with imagination exercises.

## Introduction

Burnout has been conceptualized in many different ways throughout the years (1–3) with the multidimensional theory by Maslach (4) being one of the most prominent approaches and the corresponding *Maslach Burnout Inventory* (5) the most widely used assessment tool (6). It consists of three dimensions: emotional exhaustion, depersonalization (referring to a negative or cynical attitude towards others or the job), and reduced personal accomplishment (regarding self-efficacy, competence, and productivity). In the case of therapists, burnout can negatively affect professional effectiveness as it can lead to more errors in judgment and less capacity for showing empathy (7, 8). Maintaining a strong therapeutic alliance, therefore, becomes difficult, which then compromises the treatment quality for clients.

The estimated prevalence of burnout among mental health professionals ranges widely from 21% to 67% depending on the studied population, country, and operationalization of burnout (9). Despite this wide range, studies consistently demonstrate that a significant percentage of mental health professionals, including psychotherapists, suffer from burnout. For instance, a meta-analysis and systematic review of 62 studies across 33 countries by O'Connor et al. (10) found that approximately 40% of mental health professionals experience professional burnout. Another systematic review, which focused specifically on psychotherapists, included 40 articles published between 1986 and 2016 and showed a 54.54% prevalence for moderate to high levels of burnout among therapists (11). More recently, Spännargård et al. (12) conducted a study on psychotherapists in Sweden, measuring burnout with the Copenhagen Burnout Inventory (CBI; 13). Of the 327 therapists, 50% reported moderate levels of burnout symptoms and 12% reported high levels, demonstrating again that over half of the included therapists experienced symptoms of burnout.

Given the high prevalence of burnout among psychotherapists and its negative impact on them and their clients, finding effective interventions to improve working conditions and enhance therapists’ resilience is crucial. However, intervention studies on mental health professionals, specifically therapists, remain scarce, as shown by a systematic review by Bell et al. (14). They identified only 15 studies that met inclusion criteria like providing an intervention regarding burnout or stress and including participants within the field of psychology. Just three of these studies were conducted with practicing psychologists as participants, which highlights the need for more intervention studies. Their findings point toward interventions that include mindfulness and self-care as a starting point for further research with an emphasis on individual differences and needs.

### Relaxation as an intervention for burnout

Relaxation techniques have emerged as a promising tool for managing stress, anxiety, and burnout with exercises and methods such as progressive muscle relaxation (intentionally tensing and relaxing muscles), mindfulness (non-judgmental focus on the present), and guided imagery (relaxing by using mental images; 15–18). They have been shown to reduce psychological stress markers like subjective anxiety and perceived stress as well as physiological markers such as cortisol levels, heart rate variability, and skin conductance levels (SCL) (19–24). Accordingly, regular relaxation practice is often recommended as a supportive treatment tool for patients alongside traditional therapy (25). Unfortunately, research regarding therapists remains scarce, with only a few studies addressing specific interventions like relaxation and mindfulness. One of these studies compared two mindfulness interventions (body-centered vs. mind-centered) for mental health professionals (26). They observed a significant decrease in anxiety, stress, and burnout measures with medium to large effect sizes (*d* = 0.70–0.95) for both interventions after engaging in the exercises. Follow-up after six months showed that significant medium to large effect sizes for stress (*d* = 0.89–0.90) and burnout (*d* = 0.63–0.86) were maintained. This underlines the potential of relaxation and mindfulness as easily accessible, low-threshold interventions for mental health professionals.

However, the duration of the interventions administered and proposed by Ruiz-Íñiguez et al. (26) exceeded five hours per week. One might argue that this kind of time allocation towards relaxation exercises may not be feasible for already stressed and time-constrained people. This raises the question of whether shorter and more flexible types of interventions could offer a more efficient yet still effective alternative for people working in the mental health system. A systematic review of mindfulness exercises including guided imagery, meditation, and other relaxation techniques for hospital staff showed that even brief interventions (<4 h per week) were able to elicit positive changes in stress, anxiety, and well-being (27). Due to the studied interventions being adapted to the participants and their unique working conditions which led to better accessibility, even interventions as short as one to five minutes were found to be effective (28, 29). Regarding the duration of relaxation exercises, Mohammed et al. (16) recommend that the effects of short interventions (5–10 min) should be studied further. As of now, however, there is no consensus on how long relaxation exercises need to last in order to have an effect. Overall, relaxation techniques have shown promising effects for managing stress and burnout and are often part of treatment plans for patients. Despite psychotherapists being familiar with relaxation practices, the effectiveness of relaxation for themselves remains poorly researched.

#### The relaxation exercise safe place

One well-known relaxation exercise is the so-called *safe place* (30)*,* which uses guided imagery to promote relaxation and emotional regulation (31). It was initially utilized in trauma therapy as a self-soothing technique to help traumatized patients manage distressing memories (32, 33). By imagining a safe and comfortable place (e.g., forest, beach, home), negative emotions like fear and anxiety can be reduced while positive ones like peace and contentment can be enhanced (31, 34, 35). The relaxation effect is achieved by focusing one's attention on the imagined environment, which leads to the activation of the parasympathetic nervous system, also known as the rest-and-digest system. In turn, it counteracts the body's stress response, which is driven by the sympathetic system (36, 37). This highlights the potential effectiveness of the safe place exercise in mitigating stress and fostering relaxation, while still being an easy-to-perform and time-efficient intervention.

However, having to create vivid mental images and immerse oneself in them raises the question of whether people with low imagery ability can still benefit from this type of intervention. The term *imagery ability* refers to one's ability to generate vivid mental images and, most importantly, to engage with them on a sensory and emotional level as if they were real (38, 39). Some studies have shown that people with higher imagery ability benefit more from guided imagery exercises when considering cortisol levels, stress, and mood (40, 41), as well as pain management in cancer patients (39). On the other hand, a more recent study, conducted with 30 students, found no significant positive impact of imagery ability (split into vividness, imagery control, and absorption) on anxiety in addition to positive and negative affect (42). As of now, research in this area remains extremely limited, with existing studies having considerable differences regarding methodology, outcome measures, and participants. Despite this, imagery ability may play a key role in the effectiveness of guided imagery interventions. Individuals with lower imagery ability might benefit to a lesser extent from such exercises. With rapid technological advancements, it may be possible to bridge this gap. By incorporating externally generated imagery via artificial intelligence (AI) and virtual reality (VR) for example, one can reduce the reliance on internal visualization abilities. This could make guided imagery effective for a wider range of individuals, regardless of their imagery abilities. Such an integrative approach was taken by Frewen et al. (43) in three proof-of-concept studies. They compared traditional imagination-based guided imagery interventions like the safe place with comparable 2-D (standard computer screen) and VR conditions. They found that participants reported better vividness and significantly higher positive affect and satisfaction in the VR interventions than in the other conditions with mostly large effect sizes (*d* > 0.80). These findings serve as a first indication that the use of such technologies might potentially enhance the exercises' inherent relaxation benefits.

### Virtual reality for relaxation

As seen in two systematic reviews with over 800 participants respectively, research regarding VR relaxation interventions has repeatedly yielded positive outcomes (44, 45). These outcomes were observed both in the general population and in individuals with mental health conditions, particularly those related to anxiety and stress. Studies indicate that relaxation interventions administered via VR lead to an immediate reduction in stress and anxiety levels, alongside increased relaxation, and positive affect (43, 46–49). Furthermore, they suggest that VR relaxation exercises are less cognitively demanding and at the same time more engaging than their traditional counterparts. All this might contribute to VR exercises' high effectiveness and improved adherence. Additionally, the positive effects of VR relaxation are even more pronounced when natural elements such as beaches, mountains, or forests are incorporated into the virtual environments (22, 44). Such natural settings have also been shown to activate the parasympathetic system, which facilitates relaxation (50).

Besides the kind of virtual environment, the concept of presence has been identified as a key factor for the effectiveness of VR relaxation (51, 52). Presence, in the context of VR, refers to the subjective experience of being present in a virtual world despite knowing that it is computer-generated (53). A stronger sense of presence can make virtual experiences feel comparable to non-simulated ones in the real world (54). Moreover, studies have found that higher levels of presence positively affect stress reduction, emotional state, and the acceptability of VR interventions (55–58). Consequently, when implementing and assessing relaxation interventions in VR, its ability to conjure a strong sense of presence should be taken into consideration as well.

Due to technological advancements opening up new possibilities, the personalization of virtual environments has emerged as another factor that could further enhance VR experiences and their effectiveness. Dai et al. (59), for example, have developed a VR program for trauma stabilization which offers personalization for users by allowing them to choose from different VR environments, lighting settings and objects to add to their own virtual safe place. A similar approach with preset options was used by Pardini et al. (60) when they conducted a study with 20 participants who received a guided body-scan relaxation intervention in both a standard and a personalized VR environment. In the personalized VR intervention participants could choose between different visual and auditory elements, and nature settings. They found that participants reported significantly higher levels of relaxation and pleasantness after the personalized VR condition compared to the standard VR condition (no effect sizes reported). Further qualitative analyses demonstrated that the personalized VR experience was preferred over the standard one. This was due to factors like a greater sense of control over the experience or a feeling of reminiscence as participants could choose VR settings they associated with personal memories. Given that the ability to personalize VR environments is rather new and requires appropriate hardware, software, and expertise, only a small number of studies have incorporated this aspect in VR relaxation research (viz., 48, 60). With these constraints in mind, AI might serve as a valuable tool in these personalization efforts (61). Without needing to rely on the user to manually select preprogrammed settings, AI can quickly adapt to various individual preferences. This approach could simplify the personalization process and simultaneously expand customization possibilities, which would make it more user-friendly, accessible, and practical for broader use.

Further, VR relaxation tailored to the needs of mental health professionals might provide a feasible intervention option in the workplace, as two studies have shown (62). Both were conducted with mental health staff in a clinic setting (*N* = 12 and *N* = 22) where participants were provided with a single-session VR relaxation intervention in a virtual nature environment for up to an hour. They found that the intervention significantly increased relaxation (large effects with *d* > 1.00 in both studies), happiness (medium to large effects with *d* = 0.63–1.48), and connectedness to nature (large effects with *d* > 0.90). Large effects were observed for the decrease in stress (*d* > 0.90) and anxiety (*d* > 0.90), and small to medium effects for sadness (*d* = 0.28–0.68). Overall, participants in both studies regarded the VR intervention as helpful, relaxing, and as a positive break from work.

Another recent study, which offered participants (mental health staff; *N* = 38) multiple 20-minute VR relaxation sessions over several weeks, found similar positive results (63). They observed a small significant increase in subjective well-being (Hedges' *g* = –0.43), a medium decrease in stress (*g* = 0.65), and small ones for burnout (*g* = 0.48) and worry (*g* = 0.46) between the beginning and end of the study. A notable aspect of this study was that participants were able to choose the time at which they completed the interventions depending on their work hours and schedules. It was emphasized that such scheduling flexibilities are an important factor for the feasibility of and adherence to these interventions with this specific work setting. It can also be considered a small step towards more user-involvement in the research process.

### The current study

The participants, consisting of psychotherapists in an outpatient setting, were involved in the planning process of the research design and were able to customize their VR experience. Such user-involvement is a crucial yet often neglected aspect of the successful implementation of VR relaxation in the workplace (64). With this approach, the users' views, values, and attitudes can be taken into consideration when planning interventions, thereby making them more responsive to users' unique needs (65). Such considerations manage to help with the feasibility and acceptability of VR interventions, which can contribute to adherence and, in turn, effectiveness (63). The study focused on a small, clinic-based sample to allow for intensive, interactive co-design of the protocol and intervention. Using a highly engaged, homogeneous group facilitated careful tailoring of the experiences and ensured comparability across participants, which is important in early-stage exploratory research where detailed user input is critical.

During a six-week-long study period, the therapists were provided with short interventions, the safe place imagination exercise and a comparable intervention in an AI generated safe place in VR. The goal of this case series was to explore their effects on relaxation, stress, and burnout as well as the influence of presence and imagery ability. This led to the following exploratory research questions (RQ):

RQ1: Do participants experience an increase in subjective and physiological relaxation levels when performing the safe place exercise regardless of the intervention condition (imagination or VR)?
RQ2: Do the participants experience a higher increase in subjective and physiological relaxation levels when performing the safe place exercise in the VR condition compared to the imagination condition?
RQ 3a: Are burnout symptoms reduced between the beginning and the end of the study?
RQ 3b: Is perceived stress reduced between the beginning and the end of the study?
RQ 4a: Is there a stronger decrease in burnout symptoms following the week after a VR condition compared to the imagination condition?
RQ 4b: Is there a stronger decrease in perceived stress following the week after a VR condition compared to the imagination condition?
RQ 5: Is higher imagery ability positively associated with the perceived relaxation effect after an imagination condition?
RQ 6: Are higher levels of presence during a VR condition associated with a greater perceived relaxation effect after the session?

## Materials and methods

The study was approved by the ethics committee of the PLUS (EK-GZ 50/2024; December 2, 2024). The study was conducted at the outpatient clinic for psychotherapy of the Paris Lodron University of Salzburg from February 25 until May 1, 2025.

### Sample

To be included in the case series, participants had to work as therapists in the outpatient clinic for psychotherapy during the duration of the study, and had to own a mobile phone with the ability to install apps as questionnaires were completed online via an app. Before starting the study, participants provided written informed consent. They received no compensation.

In total, six therapists—who already completed the psychotherapy training either in Austria or in Germany—were recruited for the study. However, one participant had to be excluded as there were too many missing data points due to not receiving push notifications for questionnaires from the smartphone app and due to prolonged sick leave, which led to scheduling issues. The final sample consisted of *N* = 5 participants. Besides participating in the study, four participants attended a meeting several months beforehand to provide input regarding the research design as part of a co-design approach (66, 67) to promote user-involvement (65). There they were informed of the purpose and general concept of the study and asked to provide their opinions on the following aspects of the research design: (a) the length of the relaxation exercise to ensure it is long enough to be effective, yet short enough to remain engaging and integrable into their workday; (b) the time at which they would prefer to receive the intervention based on their schedules and needs for such (e.g., at the beginning or end of the day or between therapy sessions); (c) the overall study design to ensure its feasibility and integrability for the participants (e.g., length of the study, number of interventions, etc.); (d) possible factors that might influence their individual experiences of stress and relaxation apart from the already selected study variables. Decisions regarding the research design were made with the participants' expertise and feedback on the aforementioned aspects in mind to ensure that their needs and preferences were met. Due to this, the length of the relaxation exercise (a) was kept short (under four minutes) and participants received the intervention at the end of their workday at the clinic (b). Further, it was decided that the length of the study would not exceed six weeks and that attending one to two intervention sessions per week would be integrable for participants (c). Lastly, participants expanded upon the study variables to include questions regarding their menstrual cycle and associated changes in mood (d). However, due to technological issues with the smartphone app, the aforementioned questions were not displayed leading to missing data for everyone except one participant.

### Study design

A within-subject design that follows principles of small sample research designs (small-N designs) was chosen to accommodate limited resources, especially regarding equipment such as HMDs for VR, time constraints, and participant availability. Small-N designs are a research approach that involves repeated interventions and data collection over several timepoints for a small number of participants (68). It can thereby function as an avenue between research and evidence-based implementation for individuals (69). Additionally, when limitations prevent the use of more common research designs this approach can still provide valuable insights e.g., when studying underrepresented populations like psychotherapists (70), as it acts as an alternative to traditional research designs. In the context of this study, more traditional research designs such as a between-subject design with experimental and control groups or a within-subject design with randomized intervention orders would not have been feasible due to time and resource limitations as well as the small number of participants. Therefore, we frame this project as a case series, which allows for the systematic description and analysis of a small number of participants of particular interest. These considerations in addition to the input from participants resulted in the following research design.

**Figure 1:**
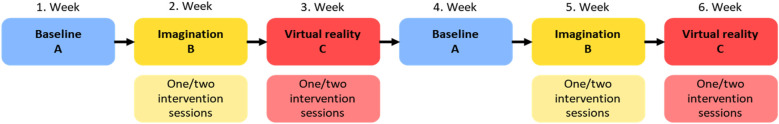
Overview of the alternating treatments study design. The study design follows an ABCABC design. During intervention weeks **(B,C)** participants received either the safe place imagination exercise **(B)** or AI-generated visualizations of the safe place in VR **(C)** depending on the week. There were no interventions during the baseline weeks **(A)**.

Participants started the study by downloading the mobile phone app *ESMira* (71). The six-week study period was comprised of baseline (no interventions) and intervention weeks where participants received the traditional safe place exercise (imagination condition) and AI-generated visualizations of the safe place in VR (VR condition) as relaxation interventions. The study followed an alternating-treatments design, a type of small-N research design, with the formula ABCABC (68). The letters stand for the phases in the study: A for baseline (no intervention), B for the imagination condition, and C for the VR condition (see Figure 1). Each phase lasted one week during which participants received either one or two sessions (during the intervention weeks) or no intervention (during the baseline weeks). The end of each study week was marked by various questionnaires (e.g., regarding burnout and stress) that were administered the day before the next week started. Intervention sessions were held at the end of the participants' respective workdays at the outpatient clinic, since the therapists considered relaxing at that time to be the most beneficial.

#### Interventions

Imagination: In the imagination condition, participants were asked to put on a pair of on-ear headphones with which they listened to an audio of a shortened version of the traditional safe place exercise. As the therapists suggested keeping the exercise short (under 5 min), already existing audio and video guides were too long. Therefore, the investigator wrote a shortened version of the safe place exercise by Luise Reddemann [see (30)] specifically for this study, which was then professionally recorded by a third party, resulting in a 3-minute and 56-second-long exercise guide. The recording starts with the instruction for the participant to close their eyes, find a comfortable position to sit in, focus on their breathing, and then start the imaginary journey to their safe place. When they arrive there, several questions are asked regarding different sensory experiences at the imagined safe place (sight, hearing, taste, smell, touch) to further intensify the imagery. After a short pause during which the participants should take in the imaginary safe place some more, the journey back to reality is initiated. The exercise ends with a couple more calm breaths to come back to the here and now [full script (German) of the exercise available in the Supplementary Materials]. SCL was recorded for the duration of the exercise.

**Figure 2:**
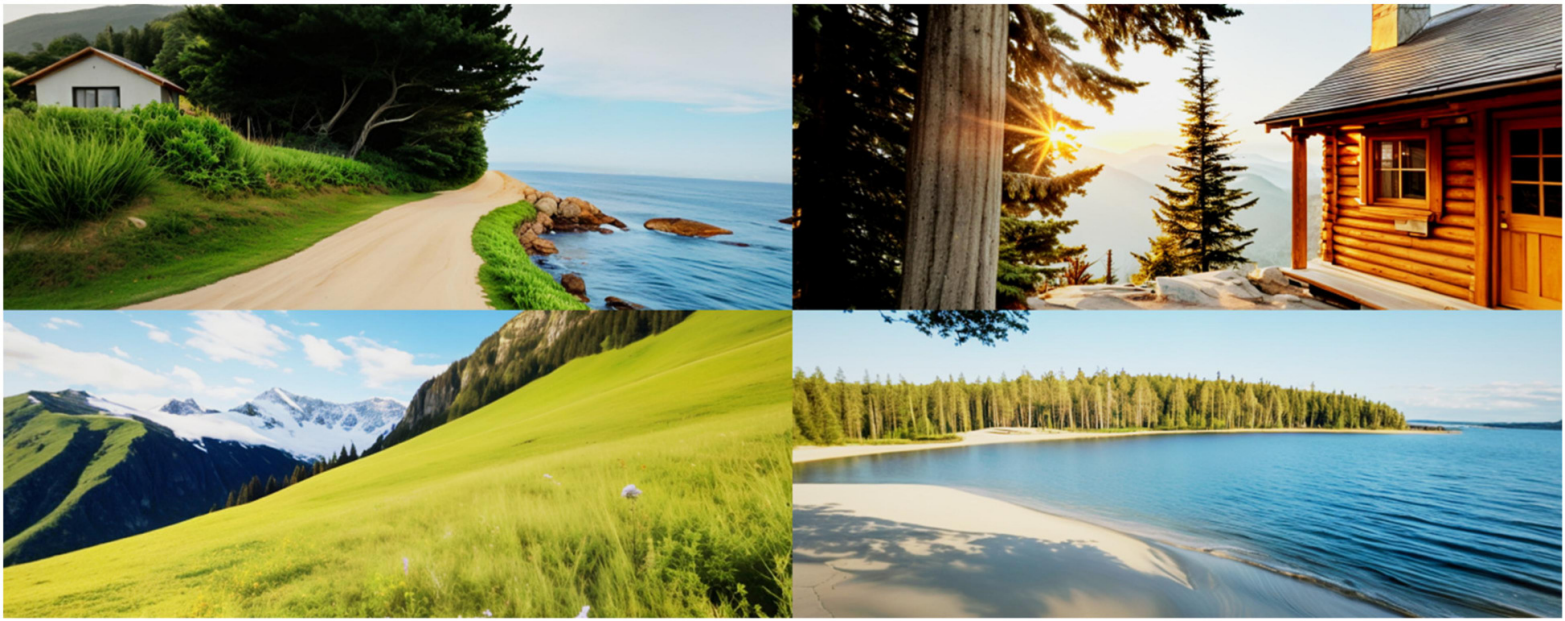
Examples of virtual reality environments. The screenshots represent examples of virtual reality environments that can be created with PsyTechVR which utilizes artificial intelligence to create virtual reality images.

VR: At the beginning of the VR condition in week 3, participants were able to create a personalized visualization of their safe place with the help of AI via the VR application PsyTechVR (72); examples of virtual safe places can be found in Figure 2). For this exercise, they were allowed to describe their safe place with a maximum of five keywords, either referring to the imagined place from week 2 or generating a new one. The investigator translated them into English for the AI software (Screenshot of the AI menu can be found in the Supplementary Material). It took approximately 30 s for AI to create the VR environment. The VR environments visualize the safe place as a static photorealistic [M3 Advanced (photo/render)] 3D scenario without sounds or movements of objects within the scenario. Then, participants determined if they were satisfied with the created environment. If the virtual environment was not satisfactory, they were able to generate a new one by changing their keywords up to two times. Next, participants put on the VR headset and headphones and listened to the safe place exercise (3 min and 56 s-long). The only difference between the VR and imagination audio guides was that the VR one instructed participants to keep their eyes open for them to get fully immersed in the virtual environment. The VR headset was the Meta Quest 3 (73), a 360° wireless HMD with a resolution of up to 2,064 × 2,208 per eye and a refresh rate between 72 Hz and 120 Hz. SCL was again recorded for the duration of the exercise. The procedure for the subsequent sessions remained the same apart from generating the safe place in VR by AI as the safe place was saved for each participant in VR in the third week to use it again in VR in the sixth week.

### Measures

The Relaxation State Questionnaire (RSQ; 74) was used before and after each intervention to measure momentary relaxation. For this study, only the general relaxation subscale (RSQ-GR) was analyzed as it offers the best fit in the context of guided imagery exercises. The general relaxation subscale is comprised of two items with high face validity and good reliability (α = .80; 74, 75). They are rated on a 5-point Likert-scale. The mean score for the general relaxation subscale was calculated with possible values between 1 and 5 and higher values indicating greater momentary relaxation.

Additionally, momentary relaxation was also assessed with a visual analogue scale (rVAS) ranging from 1 (*completely tense*) to 100 (*completely relaxed*) before and after each intervention.

Skin conductance level (SCL): As relaxation exercises like the safe place have been shown to reduce both psychological and physiological stress markers, one physiological marker was included in the study. SCL was chosen as it can provide an objective assessment of the effectiveness of the relaxation interventions. It reflects the electrical conductivity of the skin due to an increase or decrease in sweat production, which changes in response to sympathetic and parasympathetic nervous system activation (76). By that, it offers a continuous, easily quantifiable, non-intrusive, easy-to-use, and less biased measure of relaxation than self-report measures (77). In the context of the study, a decrease in SCL would suggest that the relaxation exercise effectively promoted relaxation (78). To measure SCL, participants were fitted with two circular Ag/AgCl electrodes (1.3 cm in diameter) on the middle phalanges of their index and middle fingers of the non-dominant hand during each imagination and VR session. A skin conductance electrode paste was used on the electrodes to ensure sufficient conductivity. The electrodes were connected to the *Varioport* (79) which was connected to a computer with the compatible software *VarioGraf* (80) that processed the raw SCL data. SCL was measured in microsiemens (µS) with a sampling rate of 25 Hz. Participants remained seated during the entire exercise and were instructed to reduce their movements as part of the exercise to avoid motion artifacts in the data.

Copenhagen Burnout Inventory (CBI; 81) English original by (13) was used to assess burnout over the last week and was administered weekly on the day before the next study week began. The questionnaire consists of 19 items rated on a 5-point Likert-scale with values ranging from 0 to 100 and two differing verbal response scales. The mean score can range between 0 and 100 with higher values representing more severe burnout.

Perceived Stress Scale [PSS-10 (82); original by Cohen et al. (83)]: The PSS-10 was used to assess perceived stress over the last week and was administered weekly the day before the next study week started. It is comprised of 10 items reflecting two subscales, helplessness and self-efficacy. Responses were recorded on a 5-point Likert scale and a sum score was calculated which could range from 10 to 50. Higher values indicate a greater perceived stress level.

Plymouth Sensory Imagery Questionnaire [PSI-Q (84); original by Andrade et al. (85)]: PSI-Q was used at baseline to assess imagery ability. It captures the vividness of mental images on a multisensory level including vision, sound, smell, taste, touch, bodily sensations, and emotions. The PSI-Q consists of 21 items with three items per sensory subscale. The items are rated on a 11-point Likert-scale. For this study, the mean score was calculated for all items with possible values ranging from 0 to 10 with higher values indicating greater imagery ability.

Igroup Presence Questionnaire (IPQ; 86). The IPQ was assessed after the VR sessions. It measures the sense of presence one experiences in a virtual environment. It is comprised of three subscales that assess spatial presence (sense of being physically present in the virtual environment), involvement (attention given to the virtual environment), and experienced realism (subjective experience of realism). There is a total of 14 items that are rated on a 7-point Likert scale (values between −3 and 3) with various verbal response scales. A mean score was calculated for all items ranging from −3 to 3, with higher scores indicating a stronger sense of presence in the virtual environment.

### Statistical analysis

While formal statistical testing is limited by the small sample size, descriptive patterns are presented as the main results and offer informative insights into participants' experiences.

On a participant/case level, descriptive statistics were used to summarize and visualize participants' scores, observable individual-level trends, individual data and change patterns, as well as condition-specific responses.

Furthermore, group-level analysis was conducted to address the research questions using SPSS 30.0. We applied non-parametric tests due to the small sample size and a significance threshold of α = .050. Participants could receive either one or two sessions during the intervention weeks. Three participants received one session during the intervention weeks (four sessions in total) and two participants received two sessions during the intervention weeks (eight sessions in total), so data for the additional sessions of the two participants (second imagination in week 2; second VR in week 3, fourth imagination in week 5, fourth VR in week 6) was excluded as the sample size for these sessions would have been too small to properly analyze.

For RQ1 and RQ2, SCL data was analyzed as follows: The raw SCL data for each intervention session was preprocessed on an individual level for every participant in the following steps using MATLAB (87) and its extension ANSLAB (88). First, all data that was collected before and after the intervention was removed which left the SCL data for the relaxation exercise (3 min 56 s). Then, the data was divided into four intervals of equal duration (59 s). Each 59-second interval consisted of 1,475 data points. In the next step, the data was transferred to Excel for further processing, mainly z-transformation to neutralize differences in skin conductance responsivity between participants and across interventions by standardizing SCL within the individual (22, 89). For this, the mean and standard deviation of the raw data for the whole intervention session were calculated. Each of the single data points was then z-transformed by subtracting the session mean and dividing by the standard deviation. Afterwards, the mean of the z-transformed data for each 59 s interval was calculated. In the end, this left four z-transformed SCL measurements for each intervention session that subsequently will be referred to as min 1, min 2, min 3, and min 4.

Statistical analysis for RQ1: The difference in perceived relaxation was tested by performing a Wilcoxon signed-rank test with the means of the pre-intervention RSQ-GR and the post-intervention RSQ-GR as variables for each intervention session individually. The same analyses were performed with data from the rVAS pre- and post-intervention. For the SCL, four Friedman ANOVAs were performed, one for each intervention session. There, the SCL means of the four z-transformed intervals were compared. If the Friedman ANOVA yielded a significant result, pairwise comparisons were conducted with special attention given to the comparison between min 1 and min 4 as an indicator of change in relaxation levels over the course of the exercise.

Statistical analysis for RQ2: To test the perceived relaxation effect of the imagination against the VR condition, the difference between pre- and post-intervention for the RSQ-GR was calculated for each session by subtracting the pre-intervention score from the post-intervention score. Then, the two imagination sessions and the two VR sessions were averaged separately to create mean difference scores for each condition. Lastly, a Wilcoxon signed-rank test was performed with these mean differences to compare the imagination and VR conditions. The same process was performed with the rVAS data. For the SCL, several values had to be calculated before the analysis. First, a difference score (min 4—min 1) was calculated for each of the four sessions on an individual level. The two difference scores for the imagination condition and the two for the VR condition respectively were then averaged for each participant, resulting in a mean difference score for the imagination and VR conditions respectively. To compare both conditions, a Wilcoxon signed-rank test was performed with these scores.

Statistical analysis for RQ3a/b: Separate Wilcoxon signed-rank tests for burnout and stress were conducted with the data from the beginning of the first week of the study and the end of the last week. The means of the CBI subscales and the overall CBI mean were used for burnout and the PSS-10 sum was used for stress.

Statistical analysis for RQ4a/b: Separate Wilcoxon signed-rank tests were performed for burnout (CBI overall and subscales) and stress (PSS-10). For this, data from the weekly questionnaires was used, resulting in seven time points for each variable of which only the data for the second, third, fifth, and sixth week was used. In accordance with Graham et al. (68), only data from consecutive intervention weeks (second week vs. third week; fifth week vs. sixth week) was considered in the analysis, due to the repeated measure, small-N study design. One participant had to be excluded from this analysis due to missing data for the relevant intervention weeks.

Statistical analysis for RQ5: A one-tailed Spearman correlation was calculated for each of the two imagination sessions. For this, the mean PSI-Q score for imagery ability and the difference scores (post—pre) of the RSQ-GR and rVAS were used.

Statistical analysis for RQ6: One-tailed Spearman correlations for each of the two VR sessions were calculated with the mean of the IPQ for presence and the difference scores (post—pre) of the RSQ-GR and rVAS respectively.

## Results

### Sample characteristics

The sample consisted of *N* = 5 therapists of the outpatient psychotherapy clinic. The therapists were mostly female (*n* = 4), in their early forties or fifties, held a high level of education (master's degree or higher), and had previous experiences with the safe place exercise (*n* = 4). There was considerable variation in years of work experience as a therapist (between 4 and 15 years) and the number of working hours with patients per week (between 3 and 30 h). Furthermore, three participants reported that they were already doing relaxation exercises on a regular basis and that they had prior experience with VR. A comprehensive overview of further sample characteristics can be found in Table 1.

**Table 1:** Sample characteristics: demographic, professional, previous safe place and VR experience.

Categorial variables	*n* (%)		
Gender			
Female	4 (80)		
Male	1 (20)		
Nationality			
Austrian	4 (80)		
German	1 (20)		
Highest level of education			
Master	3 (60)		
PhD	2 (40)		
Therapeutic cluster			
Humanistic cluster	3 (60)		
Behavioral cluster	2 (40)		
Previous experience with safe place			
Yes	4 (80)		
No	1 (20)		
Currently doing regular relaxation exercises			
Yes	3 (60)		
No	2 (40)		
Previous experience with virtual reality			
Yes—work-related	1 (20)		
Yes—private	2 (40)		
No	2 (40)		
Metric variables	*M*	*SD*	Range
Age (in years)	47.60	7.44	40–57
Working experience as a therapist (in years)	10.00	4.30	4–15
Work hours per week with patients	18.00	11.77	3–30
Work hours per week overall	24.00	15.17	4–38

*N* = 5. M, mean; SD, standard deviation.

#### Results for RQ1—safe place relaxation effect across conditions

**Figure 3:**
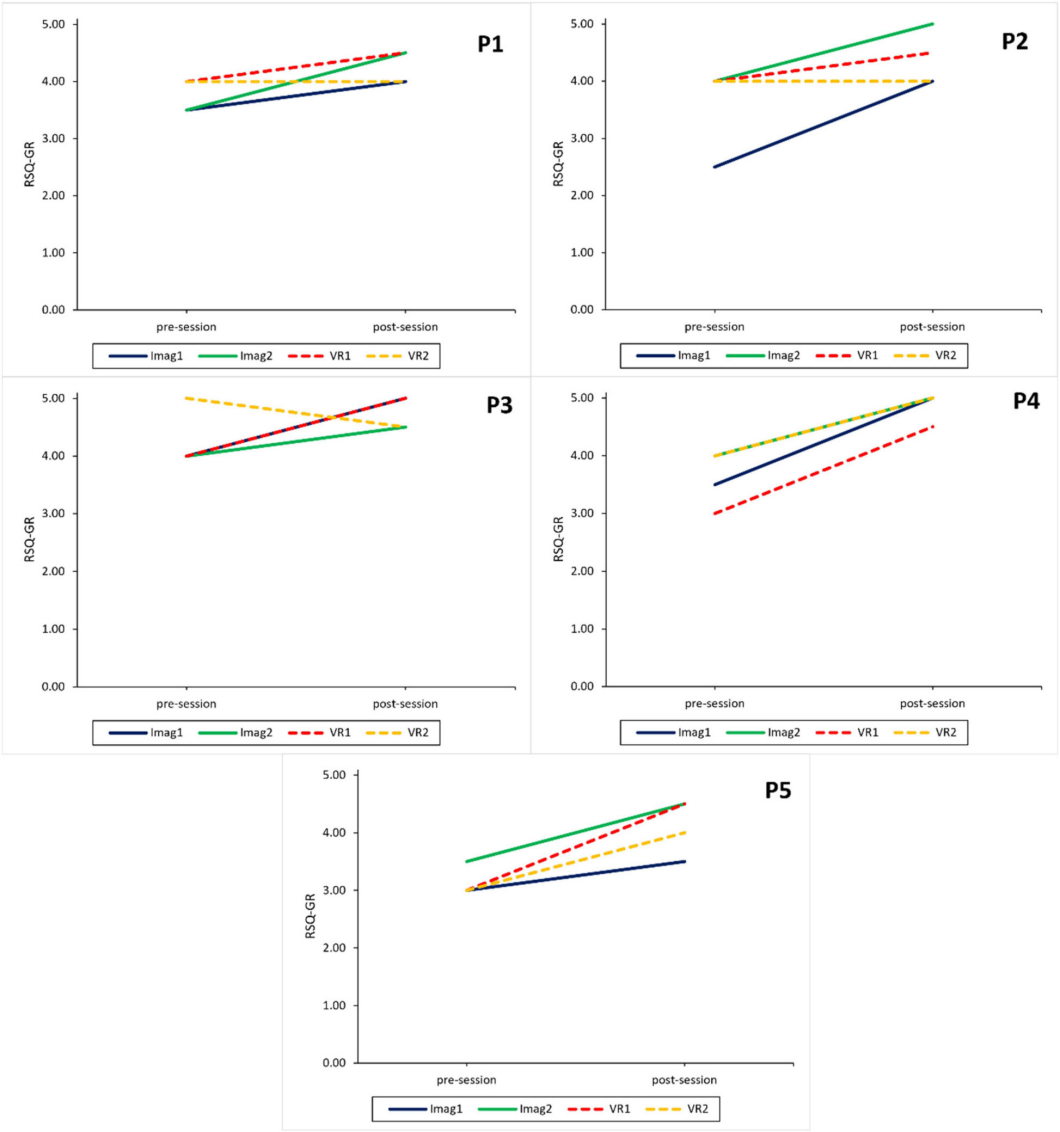
Intervention sessions: relaxation measured with the relaxation state questionnaire for each participant. RSQ-GR, relaxation state questionnaire—general relaxation subscale; Imag1, imagined safe place in week 2; Imag2, imagined safe place in week 5; VR1, virtual reality safe place in week 3; VR2, virtual reality safe place in week 6.

**Figure 4:**
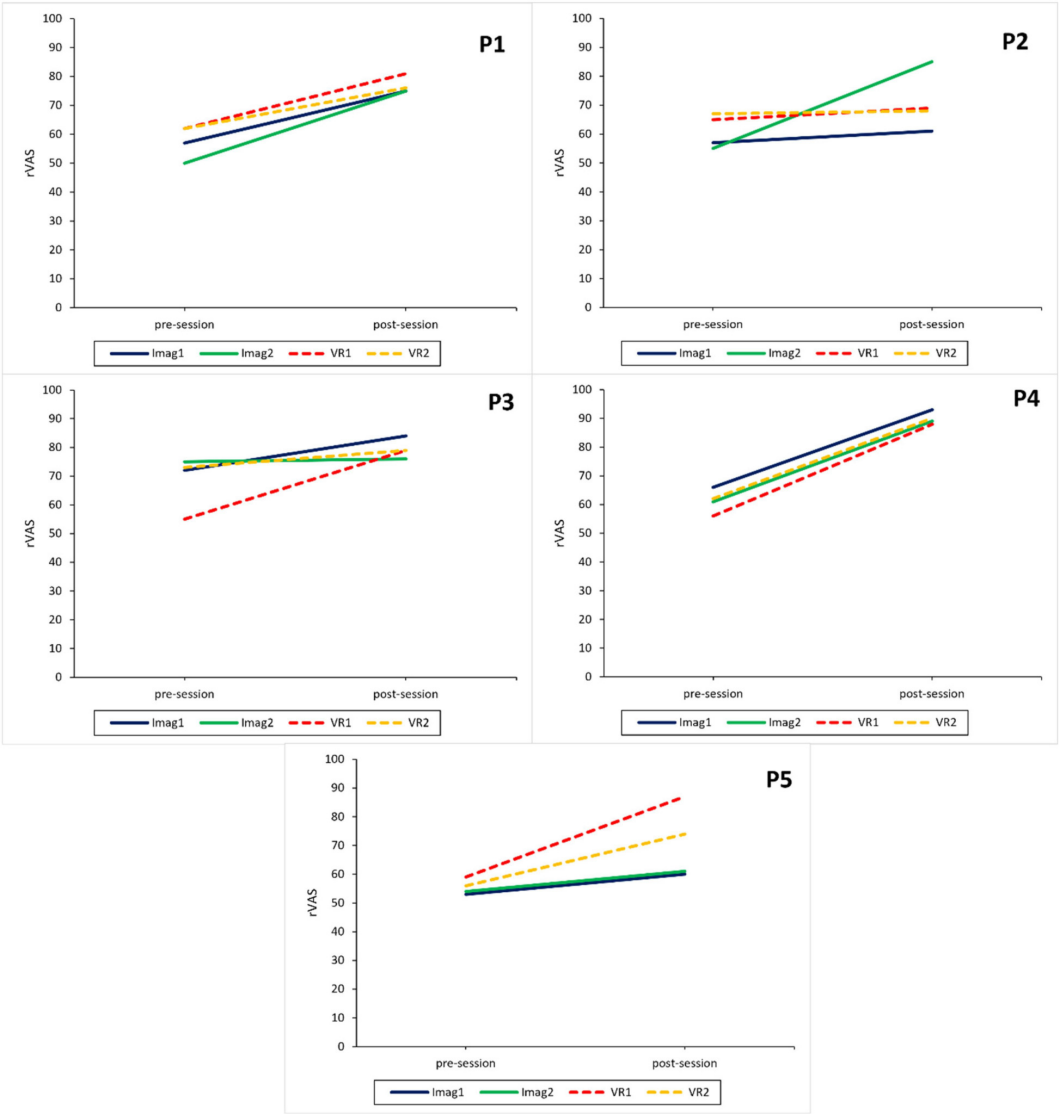
Intervention sessions: relaxation measured with a visual analog scale for each participant. rVAS, relaxation visual analog scale; Imag1, imagined safe place in week 2; Imag2, imagined safe place in week 5; VR1, virtual reality safe place in week 3; VR2, virtual reality safe place in week 6.

**Figure 5:**
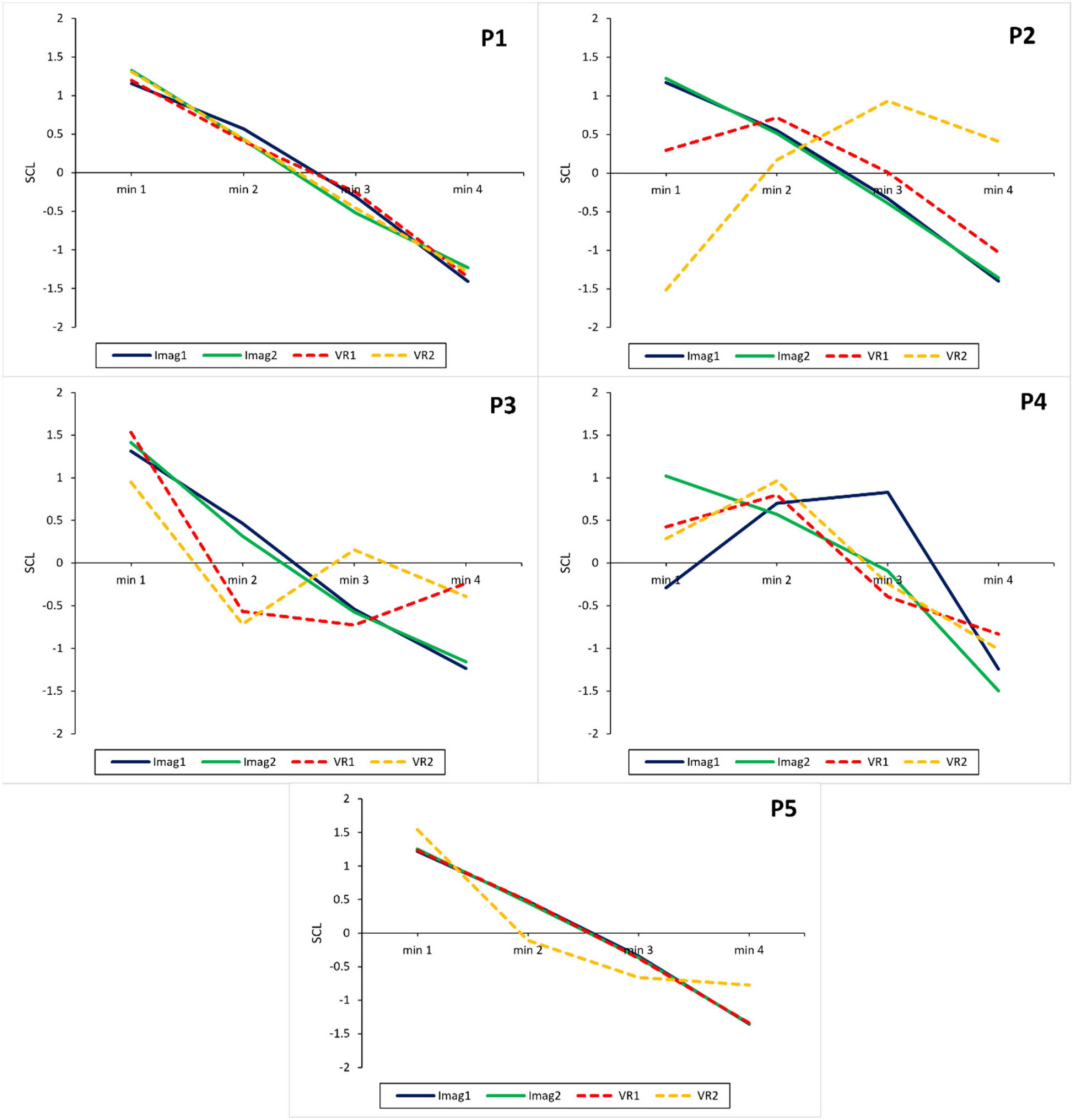
Intervention sessions: relaxation measured using skin conductance levels for each participant. SCL, z-transformed skin conductance levels. A decrease in SCL indicates an increase in relaxation. Imag1, imagined safe place in week 2; Imag2, imagined safe place in week 5; VR1, virtual reality safe place in week 3; VR2, virtual reality safe place in week 6.

On a participant level, descriptive results show that both the imagination and VR interventions lead to an increase in subjective relaxation measured with the RSQ-GR and rVAS (see Figures 3, 4) for all participants. The only exceptions were observed for the RSQ-GR during the second VR session (week 6) where participants 1 and 2 reported no change from pre- to post-session. P3 experienced a slight decrease in relaxation after starting the session with the highest possible score on the RSQ-GR (5). Further, individual SCL data (Figure 5) showed decreases across the intervention for most participants. An exception was P2 during the second VR session (week 6), where SCL started at a low point and increased over the course of the intervention. P1 and P5 showed a continuous decrease across all interventions. For P3, a marked drop between minute 1 and minute 2 was observed during VR interventions, followed by stabilization (VR1) or a subsequent increase (VR2). For P4, a slight initial increase was followed by a decrease during Imagination 1, VR1, and VR2.

Results for the Wilcoxon signed-rank test for the RSQ-GR and rVAS are displayed in Table 2. Based on the RSQ-GR participants experienced a significant increase in relaxation in all intervention sessions except for the VR session in week 6, where relaxation scores stayed roughly the same. The rVAS showed a significant increase for all intervention sessions. All significant effects were large [*r* > .50; (90)].

**Table 2:** Results of the Wilcoxon signed-rank test regarding changes in self-reported relaxation measures for each intervention session.

Relaxation measures	Pre-session	Post-session	Pre- vs. post-session		
	*Mdn.*	*Mdn.*	*z*	*p*	*r*
Imagination in week 2					
RSQ-GR	3.50	4.00	2.04	.041^*^	.65
rVAS	57.00	75.00	2.02	.043^*^	.64
Imagination in week 5					
RSQ-GR	4.00	4.50	2.12	.034^*^	.67
rVAS	57.00	75.00	2.02	.043^*^	.64
Virtual reality in week 3					
RSQ-GR	4.00	4.50	2.04	.041^*^	.65
rVAS	57.00	75.00	2.02	.043^*^	.64
Virtual reality in week 6					
RSQ-GR	4.00	4.00	1.09	.276	.44
rVAS	62.00	76.00	2.02	.043^*^	.64

*N* = 5. RSQ-GR, relaxation state questionnaire—general relaxation subscale (74); rVAS, relaxation visual analog scale; *Mdn*., median; *z*, standardized test statistic; *p*, asymptotic significance (two-sided), *r* = effect size.

*p < .050.

**Figure 6:**
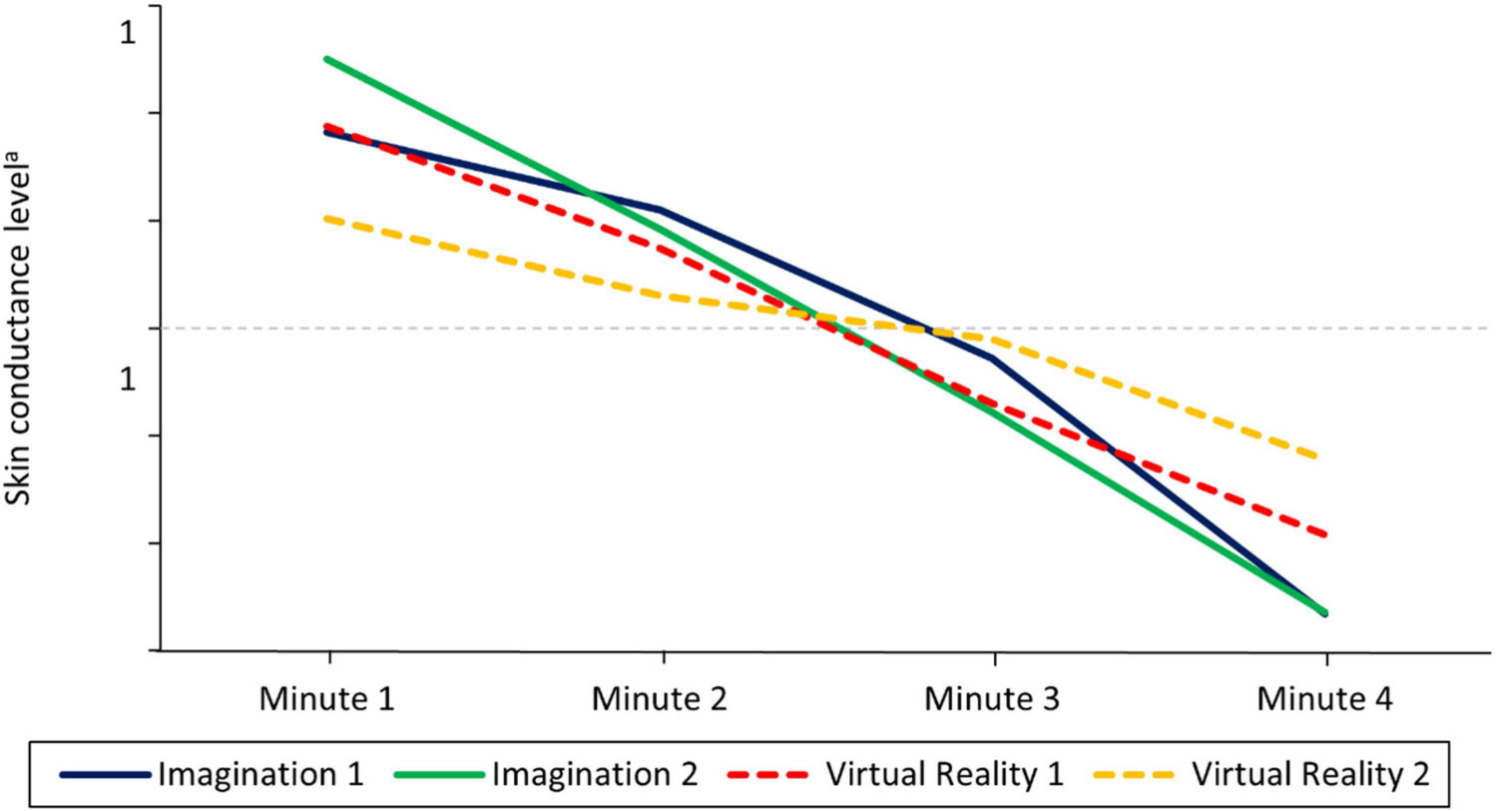
Mean skin conductance levels over the course of the relaxation exercises in each condition. *N* = 5. A decrease in skin conductance levels indicates an increase in relaxation. The figure shows how SCL decreased and thereby how relaxation increased throughout the different relaxation sessions and modalities. Z-transformed skin conductance levels are reported. Imagination 1 = Week 2, Imagination 2 = Week 5, Virtual Reality 1 = Week 3, Virtual Reality 2 = Week 6.

The Friedman ANOVA for comparing the four SCL intervals was significant for all sessions except the VR session in week 6. The decrease in SCL for all participants over the four intervals can be seen in Figure 6 while Table 3 shows the comparison between the first and last SCL intervals during the sessions. Except for the VR session in week 6, SCL significantly decreased over the duration of all other exercises and between their first and last intervals with a large effect. After further inspection of the SCL data for the VR session in week 6, it became apparent that one participant showed an increase in SCL. This was unusual for the participants as all other sessions led to a decrease in SCL. Therefore, the analysis for the VR condition in week 6 was conducted again and the participant was excluded. Due to this, a significant change between the first and last interval was observed (*z* = 2.74, adj. *p* = .037, *r* = .87), however, the other intervals did not differ significantly, and the overall Friedman ANOVA yielded only a non-significant trend [*χ²* (3) = 7.80, *p* = .050].

**Table 3:** Results of the friedman ANOVA *post-hoc* tests regarding the change of skin conductance levels between the first and last minute of the exercise.

Sessions	Min 1	Min 4	Min 1 vs. Min 4	
	*M*	*M*	*z*	adj. *p*^a^
Imagination in week 2	0.91	−1.33	3.18	.009^*^
Imagination in week 5	1.25	−1.32	3.67	.001^*^
Virtual reality in week 3	0.94	−0.96	2.69	.042^*^
Virtual reality in week 6	0.51	−0.61	1.96	.300

*N* = 5; *z*, standardized test statistic.

*p < .050.

a Adjusted by the Bonferroni correction for multiple tests.

#### Results for RQ2—safe place relaxation effect between conditions

On a participant level, overall questionnaire data regarding subjective relaxation shows similar positive changes after imagination and VR condition throughout the experiment. The effect of the VR condition appears to be weaker for the second VR session witch one participant even reporting worse relaxation scores after the intervention. No such decrease was observed for the imagination conditions (see Figures 3, 4).

The results of the Wilcoxon signed-rank tests showed that the VR condition (RSQ-GR: *Mdn.* = 0.25; rVAS: *Mdn.* = 16.50) and the imagination condition (RSQ-GR: *Mdn.* = 0.75; rVAS: *Mdn.* = 16.50) did not significantly differ from one another regarding increases in relaxation (RSQ-GR: *z* = −1.13, *p* = .257, *r* = −.40; rVAS: *z* = 0.41, *p* = .686, *r* = .13). The comparison of the mean difference (min 4—min 1) of the SCL for the imagination (*Mdn.* = −2.56) and VR condition (*Mdn.* = −1.56) with the Wilcoxon signed-rank test also showed no significant effect for the conditions (*z* = 1.75, *p* = .080, *r* = .55).

#### Results for RQ3a/b—study effect on burnout & perceived stress across conditions

**Figure 7:**
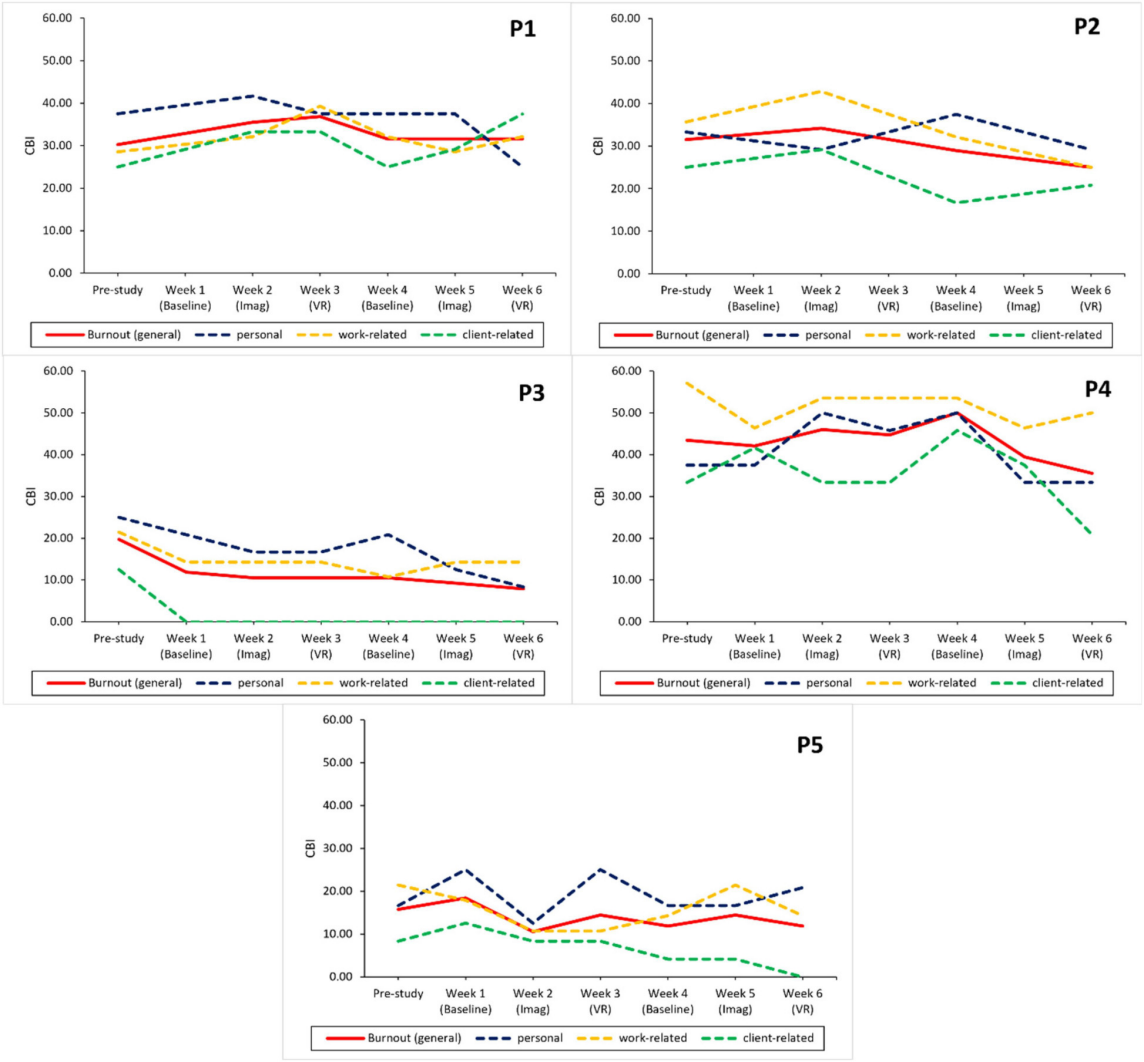
Study effects on burnout across conditions for each participant. CBI, copenhagen burnout inventory; personal, personal burnout; work-related, work-related burnout; client-related, client-related burnout; Imag, imagined safe place; VR, safe place in virtual reality. *Missing data for the study week. The line is continued to the next available data point.

**Figure 8:**
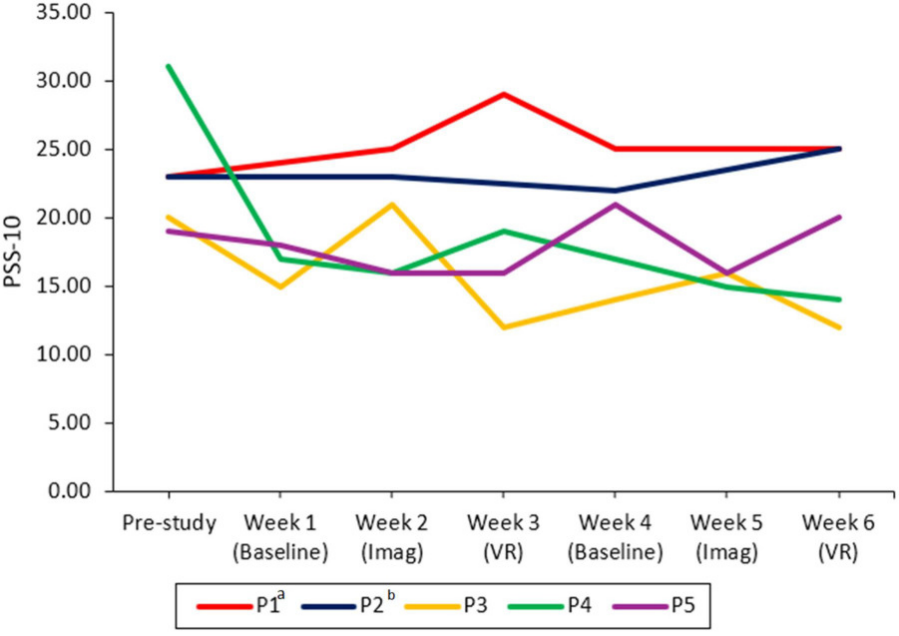
Study effects on stress across conditions for each participant. PSS-10, perceived stress scale; Imag, imagined safe place; VR, safe place in virtual reality. ^a^Missing data for week 1. The line is continued to the next available data point. ^b^Missing data for weeks 1, 3, and 5. The line is continued to the next available data point.

Descriptive trends show a slight decrease of overall burnout between the start and end of the study for all participants except P1 (see Figure 7). Personal burnout decreased for everyone except P5, and work- and client-related burnout for all participants except P1. Descriptive inspection of the data showed that only P3 and P4 experienced a decrease in stress over the study period (see Figure 8).

The four Wilcoxon signed-rank tests conducted did not yield significant results regarding overall, personal, work-related, and client-related burnout (CBI) as well as stress (PSS-10; see Table 4).

**Table 4:** Results of the Wilcoxon signed-rank tests comparing pre- and post-study burnout and stress.

Measures	Pre-study	Post-study	Pre- vs. post-study		
	*Mdn.*	*Mdn.*	*z*	*p*	*r*
Overall burnout	30.26	25.00	−1.75	.080	−.55
Personal burnout	33.33	25.00	−1.51	.131	−.48
Work-related burnout	28.57	25.00	−1.79	.074	−.57
Client-related burnout	25.00	20.83	−0.96	.336	−.30
Stress	23.00	20.00	−0.41	.684	−.13

*N* = 5; *Mdn*., median; *z*, standardized test statistic; *p*, asymptotic significance (two-sided); *r*, effect size. Burnout was measured with the CBI (Copenhagen burnout inventory (81); and stress with the PSS-10 (Perceives stress scale; (82).

#### Results for RQ4a/b—study effect on burnout & perceived stress between conditions

As one participant (P2) had missing data for the relevant intervention weeks, they had to be excluded from further analysis. The following analyses were therefore conducted with *n* = 4 participants.

Descriptive analysis for each participant showed no consistent trends for stress and overall, personal, work- and client-related burnout between the second (imagination) and third (VR) week as well as between the fifth (imagination) and sixth (VR) week. While some participants showed a slight decrease in (burnout and) stress scores (e.g., P2 & P4) other participants indicated stable scores (P1 & P2) or varying scores around a personal average (P5).

Group level analysis indicated no significant difference in all burnout scores and stress between weeks following an imagination intervention and weeks following a VR intervention. Results are reported in Table 5. Some comparisons could not be assessed as there were only tied values. For those cases (Week 2 vs. 3: personal, client-related burnout, stress; Week 5 vs. 6: work-related burnout) the test holds no meaningful interpretation.

**Table 5:** Results of the Wilcoxon signed-rank tests for consecutive intervention weeks regarding burnout and stress.

Measures	Week 2	Week 3	Week 2 vs. 3		
	Imagination	VR			
	*Mdn.*	*Mdn.*	*z*	*p*	*r*
Overall burnout	23.03	25.66	0.82	.414	.33
Personal burnout	29.17	31.25	0.00^a^	–	–
Work-related burnout	23.21	26.79	1.00	.317	.71
Client-related burnout	20.83	20.83	0.00^a^	–	–
Stress	18.50	17.50	0.00^a^	–	–
	Week 5	Week 6	Week 5 vs. 6		
	Imagination	VR			
	*Mdn.*	*Mdn.*	*z*	*p*	*r*
Overall burnout	23.03	21.71	−1.60	.109	−.65
Personal burnout	25.00	22.92	−0.82	.414	−.33
Work-related burnout	25.00	23.21	0.00^a^	–	–
Client-related burnout	16.67	10.42	−0.54	.593	.22
Stress	16.00	17.00	0.27	.785	.11

*N* = 4; *Mdn*., median; *z*, standardized test statistic; *p*, asymptotic significance (two sided); *r*, effect size. Burnout was measured with the CBI [Copenhagen burnout Inventory; (81)] and stress with the PSS-10 [Perceives stress scale; (82)].

a Due to only tied values, no statistical difference could be assessed.

#### Results for RQ5—association of imagery ability & perceived relaxation for imagination condition

The current sample was mostly homogenous regarding imagery ability except for P5 who reported having low imagery ability as can be seen in Table 6. The impact of such low imagery ability might be visible in Figure 4 where relaxation was measured with the rVAS. It shows that the two VR conditions led to a higher increase in relaxation post session than the two imagination sessions despite similar relaxation score pre session. The RSQ-GR shows a similar trend (see Figure 3), albeit not as clear as the rVAS.

**Table 6:** Imagery ability and presence scores for each participant.

Participant	Imagery ability	Presence	
		Week 3 (VR)	Week 6 (VR)
P1	6.76	0.36	0.79
P2	6.10	0.43	0.07
P3	8.24	0.79	1.29
P4	8.90	0.93	1.07
P5	2.00	0.79	0.36

Imagery ability was assessed with the PSIQ (84). Presence was assessed with the IPQ (86).

Further, imagery ability was not significantly correlated with the mean difference (post—pre) of the RSQ-GR in both imagination sessions [Imagination in week 2: *r_s_*(3) = .47, *p* = .210, one-tailed; Imagination in week 5: *r_s_*(3) = −.35, *p* = .280, one-tailed]. Further, there was no significant correlation between imagery ability and the mean difference of the rVAS in both sessions [Imagination in week 2: *r_s_*(3) = .80, *p* = .052, one-tailed; Imagination in week 5: *r_s_*(3) = .00, *p* = .500, one-tailed].

#### Results for RQ6—association of presence in VR & perceived relaxation

Presence scores were homogenous for both VR sessions with no significant outliers as the scale ranges from −3 to 3 and scores close to 0 indicate mediocre presence and no descriptive trends were observable (Table 5).

Spearman correlations yielded significant results with large effects [*r* > .50; (90)] between presence and the relaxation effect for the VR session in week 3 [RSQ-GR: *r_s_*(3) = .89, *p* = .021, one-tailed; rVAS: *r_s_*(3) = .87, *p* = .027, one-tailed]. For the VR session in week 6, however, presence and the relaxation effect were not significantly associated [RSQ-GR: *r_s_*(3) = −.32, *p* = .302, one-tailed; rVAS: *r_s_*(3) = .30, *p* = .312, one-tailed].

### Further exploratory, qualitative and descriptive results

As this study represents a novel approach by incorporating new technologies and by providing interventions for psychotherapists, a largely understudied population, it can offer valuable insights. Therefore, other variables that were not relevant to RQ1-6 were also analyzed to gain a better understanding of the technologies, interventions, and the therapists' opinions.

During the first VR session, participants generated their safe place by using up to five keywords and had the opportunity to change them two more times. Three out of the five participants used another attempt after generating the VR environment the first time by altering one of the keywords. Some themes regarding popular safe places and keywords emerged: water/ocean/beaches, forests, meadows, mountains, houses like cabins and cottages, and atmospheric or season-related keywords (warm, autumn, morning). After the first VR session, participants were asked several questions regarding their satisfaction with the VR environment. On a VAS from 1 to 100, they indicated the extent to which the AI-generated image matched their expectations. On average, they reported that the image matched their expectations to a high degree (*M* = 75.40, range = 66–100). The quality of the image was also regarded as high (*M* = 73.40, range = 59–79). Overall, participants were highly satisfied with the generated images (*M* = 80.20, range = 70–100). They were also asked to give their opinions on what they would change about the image if they had the opportunity. Participants would have liked to add sounds and motion to their VR environment or change details like the positioning of trees or the number of mountains.

After the second imagination intervention, participants were asked whether they imagined the same safe place as in the first imagination session, a different one, or if they thought of the VR environment from two weeks prior. Two participants imagined the same safe place (P2, P4), two conjured a different one (P1, P5), and one participant thought back to the VR environment (P3).

At the end of the study, participants were asked if they would recommend VR for relaxation to others. Three participants would recommend it to colleagues (P1, P4, P5), four to their patients (P1, P3, P4, P5), and three to their friends and/or families (P2, P4, P5). When asked whether they would continue VR relaxation, three participants answered *yes* (P2, P4, P5) and one *no* as they did not enjoy VR (P3). Another participant (P1) gave direct feedback that they were already satisfied with their regular relaxation exercises and would like to use VR for other things like gaming or for educational purposes. However, there were no observable trends regarding the effectiveness of the VR sessions for individuals who disliked VR.

## Discussion

The primary aim of the current exploratory case series study was to explore the effect of a brief relaxation intervention and to investigate differences between the traditional safe place guided imagery exercise with its VR adaptation in a small sample of psychotherapists.

First, the findings mostly suggest that performing the safe place exercise led to increased relaxation levels regardless of the intervention condition (RQ1), indicating that both the imagination and VR conditions could enhance relaxation among therapists. This was shown by consistent improvements on the rVAS in all sessions and the RSQ-GR in most sessions. Further, SCL also declined throughout most sessions, indicating increases in relaxation. These results are consistent with prior research that demonstrated the effectiveness of guided imagery techniques as well as VR-based relaxation [e.g., (31, 35, 43, 44)]. Only the VR session in week 6 did not yield consistent positive results when relaxation was measured with the RSQ-GR and the SCL. As already mentioned in the results, the SCL measurement for one participant was unusual as it noticeably differed from the rest in that session and from other measurements from this participant. This could be due to measurement errors, artifacts in the data such as movement, or unidentified confounding variables that influenced the SCL. When the same group-level analysis was conducted without this participant, the results showed that the SCL significantly decreased from the first to the last interval as expected. However, the decrease across all intervals failed to reach statistical significance by a narrow margin. As for the RSQ-GR as a measure of the effectiveness of the VR session in week 6, it showed no increase in relaxation as the scores remained relatively stable. This lack of change could be attributed to ceiling effects given that relaxation levels were already high before the intervention (*Mdn.* = 4.00; Scale maximum = 5.00), which left little room for measurable improvement. In contrast, the rVAS may have been less susceptible to such ceiling effects as it provided a larger and more nuanced response scale. Additionally, as the same VR environment was used for the VR sessions in week 3 as well as in week 6, there could have been a habituation effect which could explain why the VR session in week 6 did not lead to significant increases in relaxation. Contrary to this, during imagination sessions, participants were able to change and adapt their imaginary safe places throughout the session as well as across sessions in week 2 and week 5. This could have allowed for novelty and better personal relevance in the moment, thereby sustaining the relaxation effect throughout the imagination session in week 5 but not the VR session in week 6. Overall, however, group-level results should be interpreted with caution as significance is highly dependent on individual data points which can be seen with the outlier in the SCL data due to the small sample size and low statistical power.

It should be kept in mind that the sample consisted of psychotherapists who were mostly familiar with the safe place exercise (*n* = 4) and relaxation practices in general. It is possible that the use of such a well-established method may have additionally contributed to participants' positive responses. The familiarity may have reduced the cognitive demand needed for the imagination exercise as participants did not have to learn and familiarize themselves with a completely new technique. Further, their prior experience might also have allowed them to enter the exercise, their safe place, and a calming mental state in general more smoothly and with less mental effort.

Furthermore, the present findings for RQ1 suggest that even short interventions, in this case approximately four minutes long, are likely sufficient to elicit measurable positive changes in relaxation and stress levels. This is in line with previous research showing that interventions between one and five minutes were effective (28, 29). Especially in the context of workplace relaxation such short interventions are more feasible and easier to integrate into the workday without disrupting it entirely. This is also backed by feedback from participants regarding the study design, as they requested a short exercise (under five minutes).

Exploratory analyses did not indicate a consistent advantage of the VR condition over the imagination exercise in promoting relaxation (RQ2), although both appeared to elicit similar responses. This stands in contrast with previous research that reported superior effects of VR compared to traditional interventions on outcome variables such as relaxation, affective state, heart rate, etc. [e.g., (43, 48)]. However, a recent study also found no significant difference for promoting relaxation between a VR condition and a traditional relaxation exercise [progressive muscle relaxation (PMR); (91)]. Their findings suggest that VR is as effective in eliciting relaxation as the conventional method and that VR might not substantially improve upon the relaxation effect. The results of the current study can be interpreted in a similar way, however, several factors may have influenced the results and should be taken into account. As already discussed, participants in this study were psychotherapists who were already familiar with the imagination exercise which could have reduced the cognitive demands necessary to perform the imagination exercise. One advantage of VR interventions is that they are less cognitively demanding than their traditional counterparts (46) as they are able to provide a relaxing environment without using cognitive resources which would be needed for imagination interventions to create vivid mental images. This relative advantage of the VR condition over the imagination condition may have been mitigated by the participants' prior experience and familiarity with the imagination exercise. This has likely made the imagination condition equally accessible and effective for them. Furthermore, although imagery ability did not seem to be associated with the effectiveness of the exercises in this study, research regarding these influences remains limited. For instance, individuals with high imagery ability might be better able to immerse themselves in imagination exercises and thereby benefit more from imagination exercises (39–41). On the other hand, those with low imagery ability may benefit more from VR as it offers external guidance and sensory input which could compensate for weaker imagery skills. However, as the role of imagery ability in the effectiveness of guided imagery exercises remains inconclusive due to mixed study outcomes [cf. (42)], further research is necessary in this regard. Lastly, personal preferences regarding relaxation modalities might also play a role, especially regarding the use of and adherence to relaxation in everyday life as seen with one participant who explicitly expressed that they would rather use VR for other purposes such as gaming or education (P1). However, this participant did not seem to benefit less from the VR sessions than the others based on descriptive analysis (see Figures 3, 4). The study by Guillen-Sanz et al. (91), where they compared VR and PMR briefly addressed such individual preferences. As one participant was unable to participate in the conventional guided PMR training due to anxiety-related symptoms which made in-person training uncomfortable, VR offered a better suited alternative to this participant. Such individual limitations/preferences which would normally restrict access to guided relaxation trainings could be mitigated with the use of VR. As research addressing individual preferences remains rare, future studies should examine how they might influence the effectiveness of different interventions.

Contrary to expectations (RQ3a/b), the data suggest burnout and stress levels remained largely stable over the course of the study. Additionally, VR sessions did not appear to lead to greater improvements in these measures compared to the imagination exercises in the week following an intervention session (RQ4a/b). While these results might indicate a lack of effectiveness, they should rather be interpreted as inconclusive, as various factors might have influenced them. First, baseline levels of burnout and stress were relatively low in the present sample which left little room for significant change. As previous research [e.g., (11)] has found that around half of therapists experience moderate to high levels of burnout, the current participants did not seem to be representative in that sense. One study even used the same burnout measure (CBI), however, their results regarding the prevalence of burnout among psychotherapists (50% moderate and 12% high burnout levels) are not reflected in this study (12). This could be due to sampling effects as therapists who are already highly stressed or burned out might be less inclined to participate in a study that requires this much time, scheduling, and a lot of involvement in general. Moreover, only therapists from one outpatient clinic were included, which might have added to the sampling effect. For most therapists working at the outpatient clinic is an additional commitment on top of their regular job and responsibilities, which likely requires a high level of motivation, as well as time and emotional capacities. Therefore, therapists who already experience higher levels of stress and burnout may be less likely to take on additional workload. This kind of self-selection that might have happened before recruitment for the study even started could also explain the low baseline levels of burnout and stress in the sample.

Another aspect that influenced the results was the small sample size in addition to the limited number of sessions each participant received with most receiving only four in total with even fewer sessions for each of the intervention conditions. As other studies had a higher intervention frequency e.g., a study by Martland et al. (63) among mental health staff offered five VR sessions, the current study design might not have intervened enough to result in significant changes. While time constraints as well as availability of participants and technological equipment influenced these decisions regarding the present study design, future research might be able to overcome this to investigate these important issues further and yield more conclusive results.

The current study also aimed to provide insights into factors that can possibly influence the effectiveness of relaxation exercises. Imagery ability scores were mostly homogenous except for P5 who had low imagery ability. The possible effect of this was observable in the subjective relaxation data, where it seemed that P5 benefitted more from the VR conditions than the imagination conditions. Group-level results showed that imagery ability (RQ5) was not significantly associated with the relaxation effect in the imagination conditions. This could again be due to participants already being familiar with relaxation and imagination exercises as this familiarity combined with previous practice might have mitigated negative effects of low imagery ability on the effectiveness of the imagination condition. The findings of the current exploratory study are in line with a previous study that found guided imagery exercises to be effective across different levels of imagery ability (42). However, some studies have found that people with higher imagery ability benefit more from guided imagery exercises (39–41). Given the previously mixed outcomes and the limited explanatory power of the current findings, the role of imagery ability in the effectiveness of imagination exercises remains inconclusive. Moreover, the current results found a negative (though not significant) correlation between imagery ability and relaxation for the second imagination session, contrary to the expected positive association. Upon further investigation of the relevant data, it became apparent that four participants reported the same increase in relaxation, while the remaining one (P3) reported a slightly lower increase, thereby being the one to introduce the only variability in the data. The latter also scored high on the PSIQ for imagery ability, which suggests that the negative trend was disproportionally influenced by this one participant. In addition to the small sample size which limits statistical power, this result is likely coincidental.

Another factor that was investigated in the study was presence in the context of the VR interventions (RQ6). Participant-level descriptive analysis did not show any observable trends as presence scores were homogenous across both VR sessions. The group-level results showed a significant positive association between the level of presence in the virtual environment and the relaxation effect during the VR session in week 3. This is in line with previous research which highlights presence as an influential aspect in VR relaxation interventions [e.g., (56, 58)]. However, no significant relationship between the two was found for the VR session in week 6 and the RSQ-GR even showed a moderate (though not significant) negative correlation with presence. This inconsistency may be linked to already relatively high baseline relaxation levels in the second session, which left little room for measurable improvement. This could have led to a reduced possibility of finding significant correlations as the variability and possible extent of the increase in relaxation scores for this session were limited. Further, the small sample size and therefore limited statistical power makes such group-level analyses unstable and unreliable which is why results should be interpreted with caution. Lastly, the negative correlation of the RSQ-GR with presence could be explained by the results of the same participant (P3) who might have also driven the negative correlation between imagery ability and relaxation. This person scored comparably high on the IPQ for presence but experienced a slight decrease in relaxation after the second VR session whereas other participants reported either no change or an increase. Again, this suggests that the negative correlation in this case might have been coincidental, as it could be attributed to this participant in addition to the small sample size and limited statistical power.

### Limitations

First and foremost, the sample size was very small (*N* = 5), which severely limits the generalizability of the results to a broader population of psychotherapists or clinical psychologists. Moreover, because the sample was highly homogeneous, external validity is low, further restricting generalizability and does not provide robust effects or enough statistical power to detect small to moderate effects. While small-N designs can offer valuable insights, they are limited in their ability to reliably detect effects or draw broader conclusions. This can be seen with the results for RQ5 and RQ6 where effects (not significant) were probably driven by a single participant, who disproportionately influenced the direction of the correlations. Additionally, scheduling conflicts led to some participants completing more sessions than others which might have affected the outcome by adding variability.

Further, the order of intervention conditions was not randomized, also due to the small sample size to not introduce more variability. This could have led to potential order effects making comparisons between the intervention weeks less robust especially as VR weeks always followed an imagination week. In addition, the absence of a true control condition limits the interpretability of the relaxation effects, as improvements could also reflect nonspecific influences such as taking a break or the passage of time. This makes it difficult to disentangle temporal and sequence-related influences such as habituation from intervention effects. Such habituation/novelty effects might have played a role between the first and second VR session as descriptive data showed that the effectiveness of VR decreased over time. However, it is difficult to disentangle these effects as this might not have been due to the decreased novelty of VR but rather due to overall habituation/fatigue to the specific relaxation exercise. Therefore, future studies should consider including control or waitlist groups to be able to disentangle intervention effects from such order effects as well as natural temporal fluctuations. With respect to carry-over effects between intervention modalities, only one participant reported recalling the VR environment from week 3 during the imagination exercise in week 5. This suggests that carry-over between intervention was minimal, though it cannot be ruled out as a potential confounding factor. To mitigate such order and carry-over effects, future research could randomize the order of intervention or use a between-subject design with experimental and control groups.

Furthermore, as participants were aware of the study aims and were familiar with the primary investigator, they might have been consciously or subconsciously inclined to adjust their behavior or answers in line with the Hawthorne effect (92, 93) possibly enhancing effects. As our results only show an overall effect of both imaginary or VR safe place exercise, participants might have adapted their responses, but it seems they did so for both conditions. For self-reported measures it is not possible to rule out that answers are influenced by the fact that they are being survey, this would be less of a problem with physiological data. For this reason, SCL was included as an objective measure that is harder to actively alter than subjective questionnaire data. To mitigate such effects, future studies should continue to include physiological, difficult to alter measures. Additionally, randomized group designs and larger samples could combat negative influences stemming from Hawthorne effect (92, 93) or social desirability as study aims might not be as obvious and a single participant's answers would impact outcomes less.

Another limitation was the overall number of interventions participants received with only one brief relaxation exercise per week in the majority of cases. The intervention frequency may not have been enough to effectively reduce stress and burnout in addition to participants already reporting comparably low scores on stress and burnout measures.

Lastly, although the use of AI in this study offered innovative personalization of the environment, it did not fully harness VR's immersive potential, as it utilized static images rather than interactive, sensory-rich VR environments. This limitation aligns with participant feedback suggesting that the lack of interactive or dynamic elements may have reduced the perceived immersion and potential impact of the VR experience. Research indicates that such immersive environments can enhance relaxation and reduce stress and anxiety (94, 95). However, in the context of relaxation, making full use of interactive and sensory VR features might lead to more cognitive load for users (96). Further, such sensory-rich, dynamic and interactive VR environments may also foster excitement rather than relaxation, warranting a balanced approach where VR features are applied in a measured way to provide immersion while maintaining a calming and restorative experience. In addition to exploring richer forms of immersion, future studies should also include longer-term evaluations, as VR-supported mindfulness interventions have been shown to improve retention and attendance compared to non-VR formats (97). As technology continues to advance, AI may eventually enable the creation of interactive, sensory-rich and multi-sensory VR environments, opening new possibilities for personalization. In the meantime, future studies should explore more dynamic VR environments to maximize the potential benefits of VR interventions.

Even though the present study needs to be viewed with these limitations in mind, it also provides directions for future research. First, future studies should focus on addressing the mentioned limitations by conducting studies with larger samples, randomized intervention conditions or a between-subject study design, and an increased intervention frequency and duration—ideally through a large-scale, multicenter RCT with an active control condition to enhance generalizability, power, and validity. Second, the novel approaches used in this study, especially the personalization of the VR environment and the user-involvement approach, should be studied further as they might be able to positively affect the acceptability and feasibility of interventions. Research should also explore more immersive and interactive VR environments to better understand the potential impact of dynamic elements, such as movement, sound, and interaction and future studies could continue to examine individual differences, such as imagery ability, sense of presence, and personalization preference, as potential moderators of intervention effectiveness. Lastly, AI was shown to be able to generate highly satisfactory VR images. Therefore, AI should be further studied and utilized for personalization purposes as it offers a wide range of possibilities for both participants and researchers. As the current study only personalized the visualization of the safe place in VR by AI (static 3D scenario), future studies should examine effects of further options of AI-based personalization beyond visual stimuli such as sounds and movements of objects within the generated scenario.

## Conclusion

The present exploratory case series study investigated the responses to the safe place exercise (guided imagery and its VR adaptation) in relation to momentary relaxation as well as burnout and stress. While the small sample size limits generalizability, the results indicate that both intervention conditions were followed by an increase in relaxation. However, results regarding changes in stress or burnout and differences between the conditions were inconclusive due to low statistical power. Presence yielded mixed results as it was positively correlated with the relaxation effect for the VR session in week 3 only.

Due to several limitations, mainly low statistical power, statistical results should be handled with caution and seen as hypothesis-generating. Despite these limitations, however, the current exploratory study offers valuable insights into this understudied research area. Especially with burnout being a highly prevalent concern among psychotherapists, interventions are needed to mitigate its risks and effects. In this context, a user-involvement approach and personalization of VR environments with AI offers promising and novel ways to study and introduce such interventions in the workplace. Overall, the findings of the current study highlight the potential of imagination- and VR-based exercises in effectively enhancing relaxation and provide suggestions for future research.

## Data Availability

The raw data supporting the conclusions of this article will be made available by the authors, without undue reservation.
