# Supplementary material for: Exploring AI-personalized visualization of the safe place in virtual reality versus imagination of the safe place for stress, burnout, and relaxation in psychotherapists: a case series
Source: Front Digit Health. 2025 Nov 20;7:1665457. doi: 10.3389/fdgth.2025.1665457 (PMC12675480; doi:10.3389/fdgth.2025.1665457)

Supplementary materials

Safe place relaxation exercise script

Note that // indicates a pause.

Schließen Sie die Augen. (*Imagination*)/Lassen Sie den Blick auf einem Punkt in Ihrer Umgebung ruhen (*VR*). // Richten Sie Ihren Fokus auf Ihren Körper. Nehmen Sie eine angenehme Sitzposition ein und lassen alle überflüssige Muskelspannung los. // Wenn Sie soweit sind konzentrieren Sie sich für ein paar Augenblicke auf Ihre Atmung. Atmen Sie ein paar Mal ruhig ein und aus. // Lenken Sie nun Ihre Aufmerksamkeit nach innen und begeben Sie sich auf die Reise an Ihren Wohlfühlort. Der Ort, an dem Sie sich sicher, geborgen und rundum wohl fühlen. // Wenn Sie voll und ganz an Ihrem Wohlfühlort angekommen sind, schauen Sie sich in Ruhe dort um. Nehmen Sie sich einen Augenblick Zeit, um Ihren Ort mit allen Sinnen wahrzunehmen und zu erkunden. // Wie genau schaut es dort aus? Was können Sie sehen? Welche Farben nehmen Sie wahr? // Können Sie etwas hören? Welche Geräusche nehmen Sie wahr? // Was können Sie schmecken? Welche Gerüche nehmen Sie wahr? // Was können Sie an Ihrem Wohlfühlort auf Ihrer Haut spüren? Versuchen Sie die Temperatur an Ihrem Ort wahrzunehmen. // Was genau trägt dazu bei, dass Sie sich hier wohlfühlen? Nehmen Sie sich einen Moment Zeit, lassen Sie sich nieder und genießen Sie Ihren Wohlfühlort. An diesem Ort können Sie entspannen und Energie tanken! // Bleiben Sie noch einen Augenblick an Ihrem Ort und genießen Sie das Wohlgefühl, die Sicherheit und die Geborgenheit, die Ihnen Ihr Ort gibt. // Verabschieden Sie sich nun von Ihrem Wohlfühlort. // Führen Sie Ihre Aufmerksamkeit wieder ins Hier und Jetzt, indem Sie sich in Ihre Sitzposition hineinspüren. // Atmen Sie nochmal ruhig ein und wieder aus und kommen wieder im Hier und Jetzt an.

Menu of MindGap AI to create a Safe Place in PsyTechVR

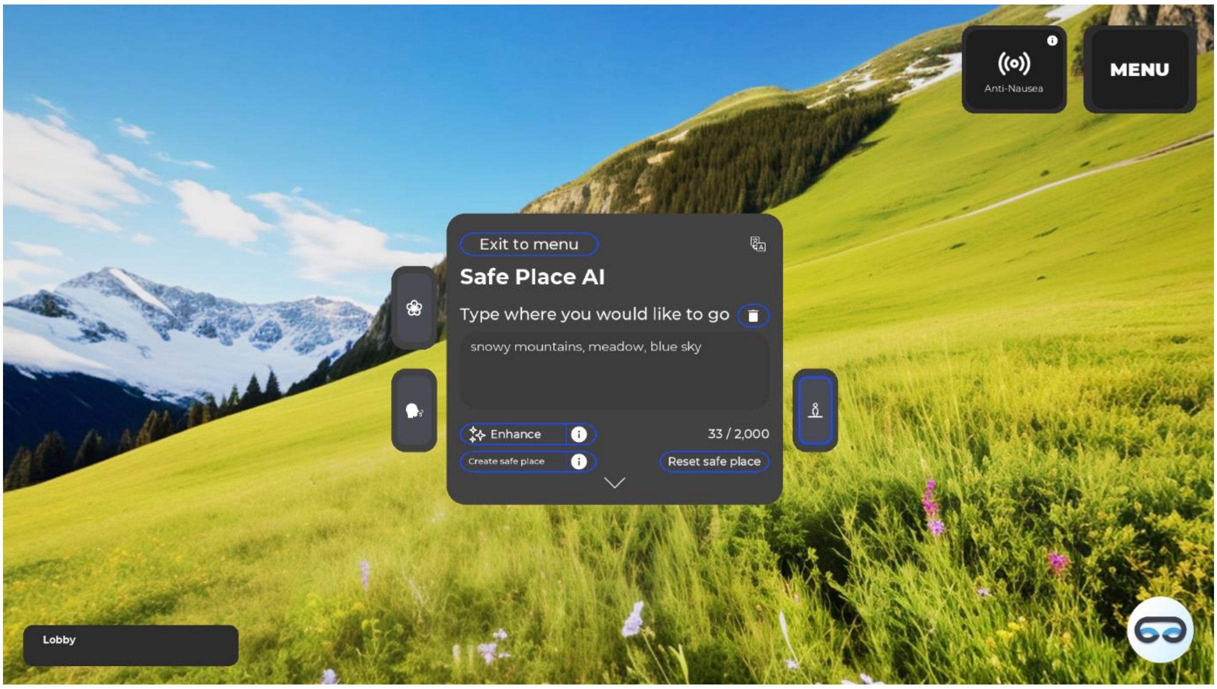

Supplement: Supplementary file 1 [file Datasheet1.pdf]
